# Activation of the left medial temporal gyrus and adjacent brain areas during affective theory of mind processing correlates with trait schizotypy in a nonclinical population

**DOI:** 10.1093/scan/nsac051

**Published:** 2022-09-15

**Authors:** Ksenija Vucurovic, Delphine Raucher-Chéné, Alexandre Obert, Pamela Gobin, Audrey Henry, Sarah Barrière, Martina Traykova, Fabien Gierski, Christophe Portefaix, Stéphanie Caillies, Arthur Kaladjian

**Affiliations:** Université de Reims Champagne Ardenne, Laboratoire Cognition, Santé, Société, EA 6291, 51100 Reims, France; Centre Rémois de Psychothérapie et Neuromodulation, 51100 Reims, France; Université de Reims Champagne Ardenne, Laboratoire Cognition, Santé, Société, EA 6291, 51100 Reims, France; Pôle Universitaire de Psychiatrie, EPSM et CHU de Reims, 51100 Reims, France; McGill University, Douglas Mental Health University Institute, 11290 Montreal, Canada; Champollion National University Institute, Cognition Sciences, Technology & Ergonomics Laboratory, University of Toulouse, 81000 Albi, France; Université de Reims Champagne Ardenne, Laboratoire Cognition, Santé, Société, EA 6291, 51100 Reims, France; Pôle Universitaire de Psychiatrie, EPSM et CHU de Reims, 51100 Reims, France; Université de Reims Champagne Ardenne, Laboratoire Cognition, Santé, Société, EA 6291, 51100 Reims, France; Pôle Universitaire de Psychiatrie, EPSM et CHU de Reims, 51100 Reims, France; Pôle Universitaire de Psychiatrie, EPSM et CHU de Reims, 51100 Reims, France; Pôle Universitaire de Psychiatrie, EPSM et CHU de Reims, 51100 Reims, France; Université de Reims Champagne Ardenne, Laboratoire Cognition, Santé, Société, EA 6291, 51100 Reims, France; Pôle Universitaire de Psychiatrie, EPSM et CHU de Reims, 51100 Reims, France; INSERM U1247 GRAP, Research Group on Alcohol and Drugs, Université de Picardie Jules Verne, 80000 Amiens, France; Radiology Department, Reims University Hospital, 51100 Reims, France; University of Reims Champagne-Ardenne, CReSTIC Laboratory, 51100 Reims, France; Université de Reims Champagne Ardenne, Laboratoire Cognition, Santé, Société, EA 6291, 51100 Reims, France; Université de Reims Champagne Ardenne, Laboratoire Cognition, Santé, Société, EA 6291, 51100 Reims, France; Pôle Universitaire de Psychiatrie, EPSM et CHU de Reims, 51100 Reims, France; University of Reims Champagne-Ardenne Faculty of Medicine, 51100 Reims, France

**Keywords:** fMRI, schizophrenia, social cognition, schizotypy, CHR, psychosis proneness

## Abstract

Schizophrenia, a severe psychiatric disorder, is associated with abnormal brain activation during theory of mind (ToM) processing. Researchers recently suggested that there is a continuum running from subclinical schizotypal personality traits to fully expressed schizophrenia symptoms. Nevertheless, it remains unclear whether schizotypal personality traits in a nonclinical population are associated with atypical brain activation during ToM tasks. Our aim was to investigate correlations between fMRI brain activation during affective ToM (ToMA) and cognitive ToM (ToMC) tasks and scores on the Schizotypal Personality Questionnaire (SPQ) and the Basic Empathy Scale in 39 healthy individuals. The total SPQ score positively correlated with brain activation during ToMA processing in clusters extending from the left medial temporal gyrus (MTG), lingual gyrus and fusiform gyrus to the parahippocampal gyrus (Brodmann area: 19). During ToMA processing, the right inferior occipital gyrus, right MTG, precuneus and posterior cingulate cortex negatively correlated with the emotional disconnection subscore and the total score of self-reported empathy. These posterior brain regions are known to be involved in memory and language, as well as in creative reasoning, in nonclinical individuals. Our findings highlight changes in brain processing associated with trait schizotypy in nonclinical individuals during ToMA but not ToMC processing.

## Introduction

Schizophrenia is a severe and often debilitating neurodevelopmental disorder that arises from an interaction between genetic and environmental factors (Kahn *et al.*, 2015). A wide range of cognitive deficits have been reported in patients (Catts *et al.*, 2013). It was in a bid to account for these complex cognitive deficits and their underlying neurobiological correlates in schizophrenia that the concept of schizophrenia spectrum disorder was developed, suggesting that the full-blown disease is only the most severe form (Guloksuz and Van Os, 2018). Converging evidence indicates that vulnerability to schizophrenia is expressed across a dynamic continuum referred to as schizotypy (Barrantes-Vidal *et al.*, 2014).

Schizotypy can be defined as a set of personality traits leading to a variety of cognitive, emotional and social behaviors that are often perceived by others to be unusual (Cohen *et al.*, 2015). It has been suggested that schizotypy is highly heritable (Romero-Garcia *et al.*, 2020), is normally distributed in the general population (Nelson *et al.*, 2013) and ranges from subclinical manifestations like social eccentricity and unusual beliefs (Cohen *et al.*, 2015) to clinical manifestations of schizotypal personality disorder (Bora and Baysan Arabaci, 2009; Ettinger *et al.*, 2014). Schizotypy is a multidimensional construct (Raine, 1991; Stefanis *et al.*, 2004; Barrantes-Vidal *et al.*, 2013), with its positive and negative dimensions being most consistently replicated (Cohen *et al.*, 2015) using a variety of assessment instruments. The Schizotypal Personality Questionnaire (SPQ) is widely used to assess trait schizotypy in nonclinical individuals (Raine, 1991), even though there has been a debate about its factor structure across cultures, with three-factor (Raine *et al.*, 1994; Dumas *et al.*, 2000; Wuthrich and Bates, 2006), four-factor (Compton *et al.*, 2009) and even five-factor (Chmielewski and Watson, 2008) models being reported. The SPQ was validated in a sample of French adults as a three-factor model (Dumas *et al.*, 2000).

The prevalence of schizotypy, defined as an expression of schizotypal personality traits, is estimated to be close to 10% (Nelson *et al.*, 2013). From the perspective of evolutionary psychiatry, this high prevalence can be viewed in terms of selective advantage (Burns, 2006). For example, some authors have suggested that creativity, defined as the ability to produce an original, innovative and context-appropriate production (Suddendorf and Fletcher-Flinn, 1997), is a potential advantage conferred by schizotypal personality traits (Park *et al.*, 2015), especially the positive dimension of schizotypy (Jacquet *et al.*, 2020; McDonald *et al.*, 2021). Furthermore, schizotypy and creativity may have a shared neural basis (Fink *et al*., 2014). Some individuals with trait schizotypy report high subjective well-being (Goulding, 2004) and invest more in leisure and creative activities, where they can excel by expressing their high level of creativity (Nelson and Rawlings, 2010). It has been suggested that individuals with marked schizotypal traits are only at risk of developing schizophrenia if they are exposed to additional risk factors (Morton *et al.*, 2017).

Schizotypy therefore provides a useful construct for studying the development of schizophrenia spectrum psychopathology, including neuroimaging markers of the disease (Nelson *et al.*, 2013). In addition, it has been suggested that schizotypy could serve as a framework for studying social cognition mechanisms in nonclinical individuals (i.e. outside the pathological context), in terms of adaptation and evolution (Cohen *et al.*, 2015).

Social cognition can be defined as the set of cognitive processes that allow individuals to understand the behaviors of others and adapt well to complex social environments (Martin *et al.*, 2014). Theory of mind (ToM) is one of the central processes of social cognition defined by the Social Cognition Psychometric Evaluation initiative (Pinkham *et al.*, 2014) and refers to the ability to attribute mental states (e.g. thoughts, beliefs, intentions and emotions) to others.

A distinction has recently been made between affective ToM (ToMA) and cognitive ToM (ToMC) at both the behavioral (Dennis *et al.*, 2013) and neural levels (Sebastian *et al.*, 2012; Bodden *et al.*, 2013; Schlaffke *et al.*, 2015). ToMC can be defined as the ability to infer the epistemic mental states of others (beliefs, knowledge or intentions) without any emotional connotation (Sebastian *et al.*, 2012). ToMA can be defined as the ability to theorize about the emotional states of others in a social context (Bensalah *et al.*, 2016).

The distinction between ToMA and ToMC brings into play the concept of empathy (Wellman and Liu, 2004; Bensalah *et al.*, 2016). Empathy is a complex neuropsychological process that enables the emotions of others to be shared, understood and responded to in a socially appropriate manner (Narme *et al.*, 2010). Three components of empathy can be identified: affective, cognitive and behavioral (Bensalah *et al.*, 2016). Emotional empathy corresponds to the process of emotional contagion, whereby the emotions of others are shared via an automatic mechanism of isomorphic emotion arousal (Narme *et al.*, 2010; Dennis *et al.*, 2013). Cognitive empathy involves processes of affective perspective-taking and emotional disconnection (Carré *et al.*, 2013; Bensalah *et al.*, 2016; Vucurovic *et al.*, 2020). Emotional disconnection refers to the ability to detach oneself from the emotions aroused by emotional contagion, whereas ToMA involves reasoning about the emotions of others. Cognitive empathy and ToMA are therefore conceptually close processes that share affective perspective-taking (Bensalah *et al.*, 2016). Finally, behavioral empathy consists in developing the social behavior most suited to the situation (Bensalah *et al.*, 2016), such as going to console a crying child. These three processes interact dynamically in individuals’ reactions to the emotional states of others (Narme *et al.*, 2010).

Impaired ToM performances have been described in schizotypy on a behavioral level (Bohec *et al.*, 2021) and in terms of brain activation during ToM tasks (Modenato and Draganski, 2015; Leung *et al.*, 2021). Results are particularly striking when high levels of schizotypal personality traits are expressed in individuals (Bora, 2020). Data from individuals at clinically high risk of schizophrenia and schizotypy suggest that ToM deficits are a holistic marker of schizophrenia spectrum disorder (Nelson *et al.*, 2013). A behavioral study conducted among a sample of nonclinical individuals with high SPQ scores found that their ToMA performances were significantly impaired, compared with those of individuals with low schizotypy traits, whereas the two groups had comparable ToMC performances (Kocsis-Bogár *et al.*, 2017).

Impaired social functioning in schizophrenia correlates with abnormal activation of the mentalizing network (Mohnke *et al.*, 2014; Kronbichler *et al.*, 2017; Jáni and Kašpárek, 2018), and several neuroimaging studies have shown that in schizophrenia there is a decrease in volume (Raucher-Chéné *et al.*, 2020) or metabolism (Dodell-Feder *et al.*, 2014; Bitsch *et al.*, 2021) in the mentalizing brain areas that are observed from the very first episode of psychosis (Bartholomeusz *et al.*, 2018). It was also suggested that impaired brain activation during mentalizing could be genetically influenced and may constitute an intermediate phenotype of psychosis (Gottesman and Gould, 2003). Recent coordinate-based meta-analyses suggested abnormal brain activation during mentalizing in individuals with a clinical high risk for schizophrenia as well (Vucurovic *et al.*, 2021). Therefore, a better understanding of brain processing during ToM in schizophrenia spectrum disorders, in particular in schizotypy-trait individuals, would help us to identify potential therapeutic targets in schizophrenia.

To our knowledge, the neural correlates of ToMA and ToMC have not yet been explored in relation to schizotypal personality traits in the general population. The aim of the present study was therefore to explore the neural correlates of schizotypal traits in nonclinical participants, while they performed a ToMA and ToMC task. We also aimed to investigate the correlation between brain activation and self-reported empathy, in order to identify how emotion-related processing of the social context might be associated with schizotypal traits. Due to the original nature of the present study, it remains difficult to state finer hypotheses. However, considering the literature, we predicted that the schizotypy dimension would be correlated with the activation of regions previously reported to be abnormally activated in schizophrenia, namely the medial prefrontal cortex (mPFC), bilateral temporoparietal junction, superior temporal gyrus and precuneus. We also expected that self-reported empathy scores would be correlated with brain activation during ToMA processing.

## Materials and methods

### Ethical statement

This study was approved by the regional ethics committee (registered under no. ID-RCB 2016-A00275-46). It was carried out in accordance with the Declaration of Helsinki, and all participants gave their written informed consent prior to being included in the study. The study was also preregistered in ClinicalTrials.gov (NTC02834182). All participants received compensation of 70 euros for their time and travel expenses.

### Participants

Here, we aimed to explore brain activation correlation with a clinical score of SPQ (trait schizotypy). Because there is a lack of available published data on this topic, it was unlikely to reliably estimate the number of participants to include before the study. Based on the data of similar design that obtain statistically significant results (Wang *et al.*, 2015; Lech *et al.*, 2016), we recruited 45 healthy individuals through advertisements in the local community. Six participants were not included in the study, two because the SPQ scale was not completed correctly and four due to functional magnetic resonance imaging (fMRI) artifacts. All participants were right-handed native French speakers with normal or corrected-to-normal hearing and vision. They had no history of neurological or psychiatric disorder according to the diagnostic and statistical manual of mental disorders 5th edition (DSM-5) (American Psychological Association, 2013) or MRI contraindication (i.e. cardiac pacemaker or other metallic implants). Participants were screened for eligibility by trained psychiatrists. The absence of a current or lifetime severe psychiatric disorder was corroborated by the Mini-International Neuropsychiatric Interview (Sheehan *et al.*, 1998).

### Experimental design

Trait schizotypy was measured with the SPQ (Raine, 1991). This 74-item self-report true–false questionnaire was originally developed to measure the nine factors of schizotypy defined by the DSM-III-R. However, a subsequent factor analytical study in an adult French population suggested that the results could be presented along just three dimensions: cognitive-perceptual, reflecting positive symptoms; interpersonal, reflecting negative symptoms; and disorganized (Dumas *et al.*, 2000). All participants completed the self-report Basic Empathy Scale (BES; Carré *et al.*, 2013).

### Experimental task

We used a previously validated cartoon task to comparatively explore ToMA and ToMC during a scanning procedure (Sebastian *et al.*, 2012).

Briefly, participants are shown a series of 30 vignette stories. For each one, they have to build a theory about the protagonists’ feelings (ToMA; 10 stories), the protagonists’ intentions (ToMC; 10 stories) or physical causality (PC; requiring an understanding of the relationship between objects, but no mental state inference; 10 stories). Each story is composed of three pictures showing two protagonists who are each introduced to the participant. Participants have to respond to the question ‘What is the end of the story?’ by deciding which of the two pictures displayed on the final screen depicts the most appropriate ending.

### fMRI analyses

The task was implemented in E-Prime 2.0 (Psychology Software Tools, Inc.) for presentation purposes and organized in a block design. Data were acquired using a Siemens 3T MRI scanner with a 20-channel head-neck coil. For each participant, a T1-weighted structural image parallel to the AC-PC line with a tilt of −30° was acquired with the following parameters: TR = 2800 ms, TE = 6 ms, flip angle = 27°, 36 slices, slice thickness = 4.5 mm, no gap, matrix = 256 × 256, FOV 240 × 240 mm^2^ and acquisition voxel size = 0.98 × 0.98 × 4.5 mm^3^. Functional data were acquired with multislice T2*-weighted echoplanar volumes with BOLD contrast. The sequence used the following acquisition parameters: 36 slices acquired using an interleaved ascending direction, same axial plane as the T1 sequence, TR = 2000 ms, TE = 30 ms, flip angle = 90°, slice thickness = 4.5 mm, no gap, matrix = 80 × 80, FOV = 240 × 240 mm^2^ and acquisition voxel size = 3 × 3 × 4.5 mm^3^. Functional data were acquired during a single run of 12 min, with 352 acquired volumes.

Imaging data were analyzed using Statistical Parametric Mapping 12th edition (SPM12) (www.fil.ion.ucl.ac.uk/spm) implemented in Matlab 2016. The first six functional image volumes were discarded to allow for T1 equilibration effects. Images were spatially realigned to the mean functional image of the series. A slice-timed correction was applied. Potential outliner scans were detected using Artifact Detection Tools algorithms. Scans showing a global BOLD signal more than five standard deviations from the mean were marked as outliers and included as nuisance regressors in first-level design matrices. Images were directly segmented and normalized to the standard anatomical space of the Montreal Neurological Institute (MNI). Resample resolution was set at 2 mm × 2 mm × 2 mm. Finally, spatial smoothing was performed with an isotropic three-dimensional Gaussian filter with a full width at half maximum of 4 mm.

First-level analyses were conducted with a modeled regressor of the three conditions: ToMA, ToMC and PC. Visual fixations and the instruction screen were modeled as variables of no interest. The realignment parameters took account of variance resulting from head movement. Data were high-pass filtered at 128 Hz to remove low-frequency drifts.

At the first level, four contrasts of interest were conducted for each participant: ToMA > PC, ToMC > PC, ToMA > ToMC and ToMC > ToMA. The resulting first-level images were then entered into separate one-sample *t*-tests in second-level analyses. Results were thresholded at *k* = 20 contiguous voxels and *P *< 0.05 familywise error (FWE)-corrected. Furthermore, correlational analyses were performed for each contrast of interest, including the total SPQ score as a covariate in the regression model. Results were thresholded at *k* = 20 contiguous voxels and *P *< 0.001. Analyses were restricted to voxels included in the mean gray matter mask calculated from individual gray matter images. Clusters of significant activation were labeled using the Automated Anatomical Labeling (AAL) toolbox (Rolls *et al.*, 2015). Significant clusters were then used in Region Of Interest (ROI) analyses. Thus, the mean activation for each contrast and cluster was extracted using the REX toolbox (https://www.nitrc.org/projects/rex/) and entered into Spearman’s correlational analyses with (I) SPQ score and subscores and (ii) BES score and subscores. All statistical analyses of behavioral data were performed using Jeffreys’s Amazing Statistics Program (JASP) (Version 0.14.1) computer software with a threshold set at *P* = 0.05.

## Results

### Behavioral data

Participants’ characteristics and SPQ and BES scores are set out in Table 1.

**Table 1.** Participants’ characteristics

	*N* = 39	
	*M*	(s.d.)
Age in years	39.51	(12.45)
Education level in years	12.33	(2.08)
BES total score	77.79	(7.59)
- Emotional contagion	19.41	(4.75)
- Cognitive empathy	33.03	(2.77)
- Emotional disconnection SPQ total score - Negative (interpersonal) - Positive (cognitive/perceptual) - Disorganized	25.36	(3.22)
	8.74	(7.54)
	3.85	(4.26)
	2.38	(2.70)
	2.51	(2.50)

Concerning task performance, the mean response accuracy was 93.59% for ToMA (s.d. = 25.53, range = 60–100), 96.67% for ToMC (s.d. = 17.97, range = 80–100) and 93.08% for PC (s.d. = 25.42, range = 60–100). There was no significant effect of condition on answer accuracy, *F*(2, 1167) = 2.81, *P *= 0.061, but a trend toward significance, as performances were better on ToMC than in the other two conditions (ToMA and PC). Mean reaction times were 3009 ms for ToMA (s.d. = 1226, range = 1722–7047), 2868 ms for ToMC (s.d. = 1126, range = 1604–7136) and 3010 ms for PC (s.d. = 1212, range = 1694–7849). There was no significant effect of condition on reaction times, *F*(2, 1167) = 1.85, *P *= 0.158.

### Functional fMRI results

**Fig. 1.**
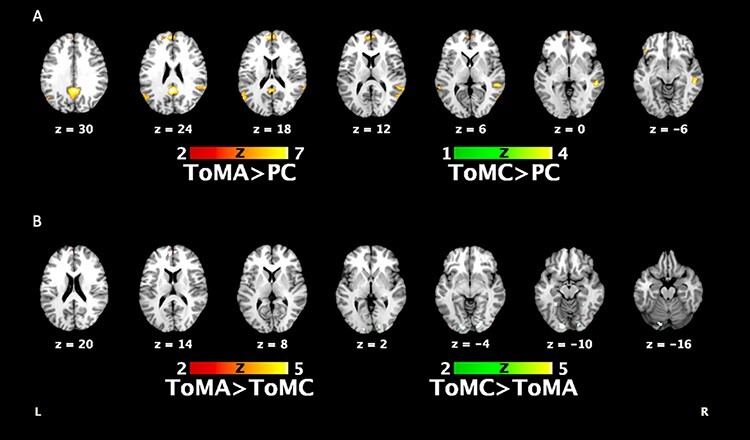
Brain activation related to the experimental task in four contrasts of interest. (A) Main effect of ToMA and ToMC relative to PC in healthy participants. Overlapping activation was observed in the precuneus. ToMA activation was widely distributed and concerned the mPFC, precuneus/PCC, superior temporal gyrus and temporoparietal junction, MTG, anterior cingulate cortex and inferior frontal gyrus. (B) The comparison between ToMA and ToMC showed that ToMA elicited the anterior part of the mPFC and the posterior cingulate (not shown), while ToMC relied on posterior brain regions (i.e. lingual and fusiform gyri). *P* < 0.05 FWE-corrected, *k* = 20.

The regions that reached cluster-level significance at *P* < 0.05, FWE-corrected, for the four contrasts of interest (ToMA > PC, ToMC > PC, ToMA > ToMC and ToMC > ToMA) are reported in Table 2 and illustrated in Figure 1. For the ToMA > PC contrast, clusters of significant activation were found in the mPFC, precuneus/posterior cingulate cortex (PCC), superior temporal gyrus and temporoparietal junction, MTG, anterior cingulate cortex and inferior frontal gyrus (Figure 1A). For the ToMC > PC contrast, clusters were found in the precuneus and medial occipital gyrus (Figure 1B). For the ToMA > ToMC contrast, clusters were found in the mPFC and PCC (Figure 1B), while for the ToMC > ToMA contrast, clusters were found in the bilateral lingual gyri and left fusiform gyrus.

**Table 2.** Whole-brain activation

Location				Significance		MNI coordinates		
Areas	Hemisphere	Brodmann area	Cluster size	*P*_FWE-corr_	*t*-values	*x*	*y*	*z*
ToMA > PC								
Precuneus	L	7	1372	<0.001	8.98	−2	−56	38
PCC	L	30		<0.001	7.65	−4	−48	22
Precuneus	L	7		<0.001	7.46	−6	−46	42
STJ/TPJ	R	22	644	<0.001	7.57	50	−20	−12
MTG	R	21		<0.001	7.35	52	−32	−4
MTG	R	22		<0.001	7.05	54	−38	6
mPFC	L	9	286	<0.001	7.39	−8	56	20
ACC	L	10		0.020	5.55	−2	58	2
SFG	L	9		0.046	5.22	−22	48	26
MTG	L	21	170	<0.001	7.18	−56	−10	−18
IFG orb	L	47	40	0.003	6.29	−42	30	−4
MTG	L	22	29	0.003	6.19	−58	−42	6
MTG/TPJ	L	19	251	0.004	6.18	−54	−62	18
Angular gyrus	L	39		0.005	6.06	−50	−64	38
STG	L	39		0.008	5.90	−54	−52	20
mPFC	R	9	164	0.004	6.16	2	56	18
mPFC	R	10		0.009	5.85	8	58	6
mPFC	R	10		0.024	5.47	2	56	0
IOG	R	19	25	0.009	5.84	46	−76	−8
MTG	R	37		0.025	5.46	52	−68	4
MTG	R	39	79	0.010	5.72	52	−58	10
ToMC > PC								
Precuneus	L	7	106	0.004	5.97	−6	−48	44
Precuneus	L	7		0.022	5.34	−2	−56	42
MTG/MOG	L	37	20	0.010	5.62	−50	−72	8
ToMA > ToMC								
mPFC	L	9	169	0.002	6.21	−6	54	22
PCC	L	31	106	0.003	6.18	−2	−46	30
mPFC	R	9	61	0.006	5.90	6	58	16
mPFC	R	9		0.037	5.20	2	52	26
ToMC > ToMA								
Lingual gyrus	L	18	202	<0.001	6.99	−14	−90	−8
Lingual gyrus	R	18	125	0.001	6.53	18	−86	−2
Fusiform gyrus	L	36	45	0.004	6.03	−26	−44	−12
Fusiform gyrus/ parahippocampus	R	37	25	0.016	5.53	28	−46	−10
Gyrus supramarginal	L	40	22	0.023	5.39	−58	−24	38

Notes. STG = superior temporal gyrus;MOG = medial occipital gyrus; ACC = anterior cingulate cortex; ; IFG = inferior frontal gyrus; TPJ = temporoparietal junction; L = left; R = right; *P*_FWE-corr_ = threshold for familywise error. *P*<0.05, FWE-corrected, *k* = 20.

**Fig. 2.**
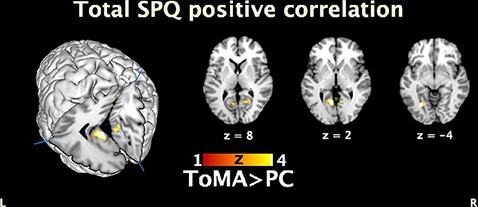
The ToMA > PC contrast of brain activation when the total SPQ was modeled as a covariate revealed a positive correlation between the total SPQ and the left posterior brain regions, namely fusiform, calcarine et parahippocampal gyrus and, in lesser extent, activation was observed in the right calcarine and lingual gyrus. On the left, oblique posterior view on the 3D reconstruction. *P* < 0.001 uncorrected, *k* = 20.

Analyses of whole-brain activation with the total SPQ score as a covariate revealed a significant positive correlation for the ToMA > PC contrast in the posterior brain regions, mostly in the left hemisphere, as reported in Table 3 and Figure 2.

**Table 3.** Whole-brain activation with the total SPQ score as a covariate

Location				Significance		MNI coordinates		
Areas	Hemisphere	Brodmann area	Cluster size	*P*_FWE-corr_	*t*-values	*x*	*y*	*z*
ToMA > PC								
Fusiform gyrus/PH	L	19	118	0.025	5.49	−30	−64	−4
IOG	L	19		0.935	3.58	−36	−70	−6
Calcarine gyrus/ PH	L	18	158	0.134	4.79	−8	−58	4
Lingual gyrus	R	18	82	0.718	3.92	10	−58	4
PCC	R	30		0.938	3.57	22	−54	8

Notes. PH = parahippocampus; PCC = posterior cingulate gyrus; L = left; R = right; *P*_FWE-corr_ = threshold for familywise error. *P*<0.001 uncorrected, *k* = 20.

### Brain–behavior associations

Four brain regions were found to be associated with clinical scores for the ToMA > PC contrast. First, the activity of the left MTG [Brodmann area (BA): 22; MNI coordinates: *x* = −58, *y* = −42 and *z* = 6] was positively correlated with the total SPQ score (rho = 0.487, *P* < 0.01), positive SPQ (rho = 0.445, *P *= 0.005) and negative SPQ (rho = 0.414, *P* < 0.01). Second, the activity of the right inferior occipital gyrus (IOG; BA: 19; MNI: *x* = 46, *y* = −76 and *z* = −8) was negatively correlated with the BES emotional disconnection subscore (rho = −0.443, *P* = 0.005). Third, the activity in the right MTG (BA: 39; MNI: *x* = 52, *y* = −58 and *z* = 10) was negatively correlated with the total BES score (rho = −0.346, *P*< 0.05). Finally, a negative correlation was found between precuneus activation (BA: 7; MNI: *x* = −2, *y* = −56 and *z* = 38) and the total BES score (rho = −0.333, *P* < 0.05).

For ToMA > ToMC contrast, activation in the left PCC (BA: 31; MNI: *x* = −2, *y* = −46 and *z* = 30) was negatively correlated with the emotional disconnection subscore of BES (rho = −0.340, *P* < 0.05).

**Fig. 3.**
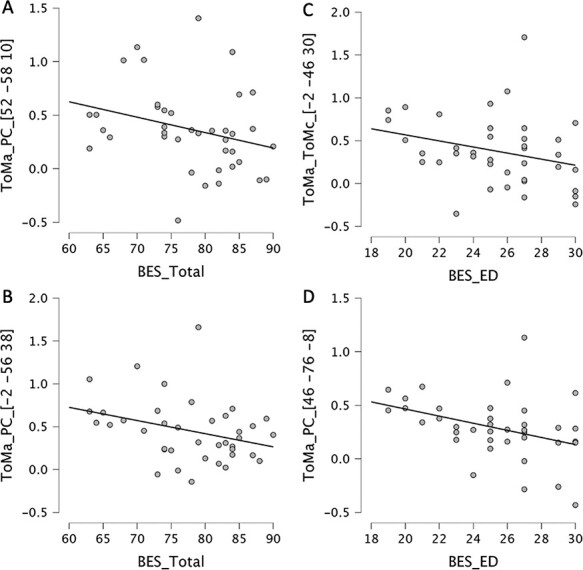
Scatter plots of significant correlations between brain activation regions for the SPQ clinical score and subscores. Brain coordinates are reported in the MNI system. In ToMA > PC contrast, we found a positive correlation between the left MTG activation and the SPQ total score (A), and positive (C) and negative (D) subscores. In ToMC > ToMA contrast, a negative correlation between the left lingual gyrus activation and the positive SPQ subscore is represented (B).

**Fig. 4.**
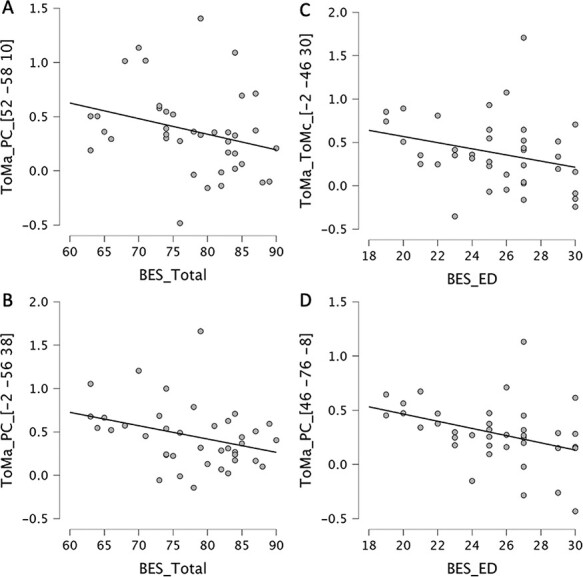
Scatter plots of significant correlations between brain activation regions for the BES clinical score and subscores. Brain coordinates are reported in the MNI system. In ToMA > PC contrast, we found a positive correlation between the total BES score and the right MTG (A), and precuneus (B). Emotional disconnection clinical subscore correlated negatively to the right IOG in ToMA > PC contrast (C) and to the PCC in ToMA > ToMC contrast (D).

A negative correlation was noted for the ToMC > ToMA contrast between the left lingual gyrus activation (BA: 18; MNI: *x* = −14, *y* = −90 and *z* = −8) and the positive SPQ (rho = −0.366, *P* < 0.05). Correlation scatter plots for SPQ scores are represented in Figure 3 and for BES scores in Figure 4.

## Discussion

In the present study, we comparatively investigated the neural bases of ToMA and ToMC in relation to trait schizotypy in a sample of nonclinical individuals. The primary goal was to show that the relationship between patterns of neural activation and trait schizotypy concerns brain structures that have previously been described as being associated with vulnerability to schizophrenia. The main finding reveals a positive correlation during ToMA processing between the total SPQ score and the brain activation in the left MTG and adjacent brain regions, namely the left lingual gyrus, fusiform gyrus and a cluster extending from the subgyral hypothalamus to the parahippocampal gyrus. Contradicting our first hypothesis, activated brain regions differed from those that are robustly reported as impaired in schizophrenia during ToM processing. One additional aim was to identify brain regions whose activation correlated with self-reported empathy and its subscores. Results suggested that the right MTG and left precuneus were negatively correlated with the total empathy score, while the left posterior cingulate and right IOG negatively correlated with the emotional disconnection subscore during ToMA processing. Therefore, the bilateral MTG seems to be involved in both trait schizotypy in the general population and emotional disconnection, the empathy component that enables emotions to be suppressed following emotional contagion while inferring the emotions of others in a social context.

As previously reported by Sebastian *et al.* (2012) in healthy adults and adolescents, the ToMA-PC contrast revealed a broader extent of brain activation that included the mPFC, a structure that has previously been reported to be critically involved in empathy and ToMA (Caillaud *et al.*, 2020). Increased activation in the posteromedial regions, including the posterior cingulate gyrus and precuneus, has consistently been reported in the literature in relation to both ToM (Schurz *et al.*, 2014) and schizotypy (Modinos *et al.*, 2010; Wang *et al.*, 2015), as well as the expression of schizophrenia-related genes (Romero-Garcia *et al.*, 2020).

The brain activation associated with the total SPQ score led to the identification of left posterior brain regions, namely the MTG, fusiform, lingual and parahippocampal gyrus, which have previously been shown to be relevant to mental imagery (Spagna *et al.*, 2021), face processing (Lobmaier *et al.*, 2008) and visuospatial cognition and memory storage (Gilbert *et al.*, 2001). Our results are in line with a number of previous studies, suggesting that abnormal left MTG functioning could serve as a marker of vulnerability to schizophrenia (Ehrlich *et al.*, 2010; Seidman *et al.*, 2014; Zhao *et al.*, 2018). Our results are also consistent with a structural MRI volumetric study in schizotypal personality disorder that found reduced cortical thickness in the left fusiform and parahippocampal gyri (Takayanagi *et al.*, 2020), providing further evidence that these regions may be associated with vulnerability to schizophrenia.

Interestingly, functional asymmetry has already been reported for face identification (Ma and Han, 2012) and language processing (Binder *et al.*, 2011). A gradual loss of gray matter in the left MTG and its surrounding brain regions has previously been observed in adolescents with schizotypal traits and mild cognitive impairment (Moorhead *et al.*, 2009).

To the best of our knowledge, this is the first study to suggest a specific role of the left MTG and its adjacent structures in ToMA processing in schizophrenia-related vulnerability.

Our results establish a link between ToMA processing, trait schizotypy and the activation of left posterior brain regions (MTG, lingual, fusiform and parahippocampal gyrus). Based on the literature, we suggest that there is a link with creativity as a facilitator of social adaptation. Creativity is defined as the ability to produce something new, original and appropriate to resolve a task and follows a U-shaped relation with schizophrenia spectrum disorder (Sampedro *et al.*, 2020). Carson (2011) described a model of schizotypy where the biological factors creating vulnerability to psychopathology confer greater creative ability on individuals, along with a higher intelligence quotient and superior metacognitive protective factors. Thus, some authors have suggested that this enhanced creativity mediates the selection of schizophrenia-related vulnerability genes through evolution (McCreery and Claridge, 2002) and explains the prevalence of trait schizotypy, evaluated at about 10% of the general population (Cohen *et al.*, 2015). In addition, ToM and schizotypy have both been related to creativity (Suddendorf and Fletcher-Flinn, 1997). Thus, trait schizotypy may promote creative reasoning, while schizophrenia may impede it (Sampedro *et al.*, 2020). Fisher *et al.* (2004) suggested that positive schizotypy is more specifically related to creative thinking, but in contrast to our findings, the authors found the underlying brain correlates in the right hemisphere, in particular the right prefrontal cortex. Divergent thinking, however, which is defined as the essence of creative thinking (Zhu *et al.*, 2013; Zhang *et al.*, 2016), has consistently been linked to inhibition (Benedek *et al.*, 2012, 2014; Zhang *et al.*, 2016). The latter is controlled by the inferior frontal gyrus (Aron *et al.*, 2014) and promoted in secondary sensory cortices for all sensory modalities processed in posterior brain regions surrounding the temporo-parieto-occipital junction (Zhang *et al.*, 2016), including the MTG. In addition, hippocampal and parahippocampal regions are involved in verbal divergent thinking (Takeuchi *et al.*, 2020). Furthermore, visual mental imagery engages the left fusiform gyrus without the early visual cortex (Spagna *et al.*, 2021), suggesting that this region plays a fundamental role in the conception of creative ideas in individuals.

The parahippocampal gyrus has been identified as a possible multimodal association area related to schizophrenia spectrum disorders in a range of studies, including postmortem research on schizophrenia (McDonald *et al.*, 2000) and brain tumors with secondary hallucinations (Acioly *et al.*, 2010), and volumetric (Seidman *et al.*, 2003; Prasad *et al.*, 2004; Moorhead *et al.*, 2009) and functional (Escartí *et al.*, 2010) MRI studies. The parahippocampal gyrus is involved in high-level cognitive functioning, including memory encoding and retrieval, as well as visuospatial cognition (Lin *et al.*, 2021). It is strongly connected to other brain regions through the inferior longitudinal fasciculus and cingulum (Lin *et al.*, 2021), both pathways that are involved in neurodevelopmental disorders, and in particular schizophrenia (Clark *et al.*, 2011). Consistent with our results, an asymmetry has been observed in structural MRI volumetric analysis, indicating that reduced gray matter volume in the left parahippocampal gyrus is specifically related to schizophrenia (Prasad *et al.*, 2004). Electrophysiological explorations using electroencephalogram (EEG) have suggested that abnormal brain activation in the left parahippocampal gyrus is specifically related to schizophrenia spectrum disorder, as it has been found in both patients with schizophrenia and their first-degree relatives, in comparison to healthy controls (Soni *et al.*, 2020). In addition, this brain region appears to be particularly vulnerable during development and is also sensitive to environmental changes, such as urbanization (Besteher *et al.*, 2017), which is thought to be the most consistent environmental risk factor for schizophrenia (van Os and Kapur, 2009).

In patients with schizophrenia, the left MTG has consistently been found to be impaired at volumetric (Marsh *et al.*, 1994), functional (Holt *et al.*, 2006) and connectivity (Zhang *et al.*, 2017) levels. It has also been associated with persistent auditory verbal hallucinations (Zhang *et al.*, 2017). Importantly, reduced gray matter volume in the left MTG has been reported in first-episode and early-onset schizophrenia (Tang *et al.*, 2012), suggesting that abnormalities in this brain region could be the most prominent marker of schizophrenia transition. Data from postmortem studies suggest that the excitatory/inhibitory molecular balance is altered in the MTG of patients with schizophrenia, mainly as a result of changes in the expression of glutamate-signaling genes (Bobilev *et al.*, 2020) and dopamine regulation (Allen *et al.*, 2019) that could further lead to positive psychotic symptoms.

Our results therefore suggest that the left MTG and adjacent brain regions display impaired activation during ToMA in healthy participants with trait schizotypy and that these brain regions should be further considered in research on the developmental aspects of the transition to schizophrenia disorder.

We should acknowledge some limitations to the study. First, we included healthy individuals with a ceiling effect on the task performance. Nevertheless, we aimed to identify how brain activation during the task would correlate with SPQ scores rather than a task performance *per se*. Second, the fMRI analyses were based on a relatively small sample that limits both the robustness of our data and the statistical application of more stringent thresholds. Therefore, the generalization of our results is limited, and this would be the first encouraging step to the other studies on the subject.

In conclusion, the present study highlights the specific changes in brain activation during ToMA (but not ToMC) processing that are associated with trait schizotypy in nonclinical individuals. These results have several implications and open up new avenues for investigating the continuum between schizotypy and schizophrenia in relation to social skill difficulties, as well as for investigating the neural brain processing related to ToM.
